# Remote Monitoring Model Based on Artificial Intelligence to Optimize DOAC Therapy: A Working Hypothesis for Safer Anticoagulation

**DOI:** 10.3390/medicina61111982

**Published:** 2025-11-05

**Authors:** Carmine Siniscalchi, Francesca Futura Bernardi, Alessandro Perrella, Pierpaolo Di Micco

**Affiliations:** 1 Internal Medicine Department, Parma Univesity Hospital, 43125 Parma, Italy; csiniscalchi84@gmail.com; 2 Department of Pharmacology, Vanvitelli University of Campania, 80100 Naples, Italy; francescafutura.bernardi@regione.campania.it; 3 First Division of Infectious Disease, P.O. Cotugno, AO dei Colli, 80131 Naples, Italy; alessandro.perrella@ospedalideicolli.it; 4 AFO Medicina, P.O. Santa Maria delle Grazie, Pozzuoli, ASL Napoli2 Nord, 80076 Naples, Italy

**Keywords:** artificial intelligence, DOACs, remote monitoring, automated alerts, anticoagulation, digital health, patient safety

## Abstract

*Background*: Direct oral anticoagulants (DOACs) have become the standard of care for preventing venous thromboembolism (VTE) and cardioembolic stroke in patients with atrial fibrillation, due to their predictable pharmacokinetics and reduced need for frequent laboratory monitoring. However, long-term DOAC use still carries a risk of complications such as gastrointestinal or occult bleeding and progressive renal decline, particularly in elderly and frail patients. *Objective*: This study proposes a remote monitoring model integrated with AI supports designed to enhance the safety and personalization of chronic DOAC therapy in both inpatient and outpatient settings. *Methods*: Building on existing national frameworks in which DOAC prescriptions are regulated by experienced physicians through regional digital platforms, we developed a structured model that integrates automatic alerts for abnormal laboratory trends, potential drug interactions, and changes in clinical status. The system uses artificial intelligence to identify high-risk patterns, such as declining hemoglobin or glomerular filtration rate, before symptoms appear, enabling early intervention. *Results*: The proposed model is presented as an integrated workflow supported by structured components. This conceptual framework facilitates real-time surveillance of patient data, supports clinical decision-making, and is expected to reduce preventable complications. Anticipated benefits include improved clinical appropriateness, better resource allocation, and reduced avoidable emergency visits. *Conclusions*: remote monitoring system integrated with AI supports for predefinite items for long term treatment with DOACs can significantly improve safety and continuity of care. By replacing passive surveillance with predictive, automated alerts, this model exemplifies how digitalization can enhance the efficiency and responsiveness of the National Health System.

## 1. Background

Direct oral anticoagulants (DOACs) have progressively replaced vitamin K antagonists (VKAs) in the management of venous thromboembolism (VTE) and the prevention of cardioembolic stroke in patients with non-valvular atrial fibrillation (NVAF), owing to their favorable pharmacological profile and ease of use. Currently, DOACs represent the standard of care for stroke prevention in NVAF and for secondary prevention in VTE cases [1,2]. Their predictable pharmacokinetics and pharmacodynamics eliminate the need for frequent INR monitoring, a key limitation of VKAs [3]. DOAC dosing adjustments are primarily indicated in cases of severe renal impairment or advanced hepatic dysfunction (Child–Pugh class B or higher). Phase III clinical trials of dabigatran, rivaroxaban, apixaban, and edoxaban demonstrated lower rates of intracranial bleeding compared with vitamin K antagonists, with variable effects on gastrointestinal bleeding [4,5,6]. These trials established the safety and efficacy of DOACs currently approved for clinical use. However, these trials typically had limited follow-up durations (12–24 months), while real-world practice often involves much longer treatment periods, especially for NVAF patients. Despite the positive safety profile observed in randomized controlled trials, long-term DOAC therapy is not without complications. Clinicians are increasingly recognizing cases of progressive anemia, often caused by occult gastrointestinal bleeding, worsening menstrual bleeding in premenopausal women, or gradual renal function decline. Many of these complications fall into the category of clinically relevant non-major bleeding, which may go unnoticed without systematic monitoring. For example, a hemoglobin drop of more than 2 g/dL, even in the absence of overt bleeding, may meet ISTH criteria for major bleeding and warrants clinical attention [7,8]. Importantly, these adverse events often go undetected due to the lack of structured follow-up and timely clinical review, especially in elderly and multimorbid patients. Delayed recognition may lead to emergency department visits, hospital admissions, blood transfusions, or even premature discontinuation of anticoagulant therapy, thereby increasing the risk of thromboembolic complications [9,10]. In Italy, DOAC prescriptions and renewals are regulated by the national drug agency (AIFA) and supported by regional digital platforms such as SANIARP and SINFONIA in Campania. These platforms integrate risk assessment tools (e.g., CHA_2_DS_2_-VASc [11] and HAS-BLED scores [12]) and laboratory data (e.g., hemoglobin, creatinine), and may also allow clinicians to report adverse events and symptoms. However, these systems are often underutilized as clinical monitoring tools and primarily function as administrative prescription gateways. Most patients are approved for 6–12 months of therapy without structured clinical reassessment [13]. This fragmented approach is compounded by long waiting lists, limited specialist access, and poor integration between electronic health records and prescribing systems. As a result, treatment decisions may be made without up-to-date clinical or laboratory data, and patients may go months without appropriate surveillance [14]. Moreover, the absence of intelligent alert systems or AI-driven analytics further reduces the capacity of clinicians to detect early warning signs, such as subtle changes in hemoglobin or glomerular filtration rate. Given these challenges, we propose a remote control of quality of anticoagulation with DOACs by remote surveillance and AI supports for predefinite relevant alerts for patients on long-term DOAC therapy. Building on current prescribing frameworks, the model incorporates artificial intelligence and automated alerts to track clinical trajectories and flag potentially high-risk trends in real time. This approach aims to bridge the gap between prescription authorization and clinical follow-up, optimizing decision-making and improving patient safety through proactive digital intervention [15]. Although DOACs include both direct thrombin inhibitors (dabigatran) and direct factor Xa inhibitors (rivaroxaban, apixaban, edoxaban, and dabigatran), our monitoring model is intended to encompass the entire class, as the risks of occult bleeding, renal function deterioration, and polypharmacy interactions are common to all agents.

### 1.1. Rationale

The growing population of patients receiving long-term DOAC therapy requires a shift from reactive, fragmented care toward a proactive, data-driven management model. Current DOAC monitoring systems in Italy and other countries rely largely on periodic administrative reassessment for prescription renewals, often without sufficient clinical context. Although regional digital platforms allow documentation of basic laboratory data and risk scores, they generally lack predictive capabilities, automated alerts, or intelligent prioritization of patients based on clinical need.

### 1.2. Objectives

The primary objective of this study is to develop and implement a structured, AI-powered remote monitoring system for patients on chronic DOAC therapy. The model is designed to fill critical gaps in the existing healthcare workflow by enabling early detection of complications such as occult bleeding, progressive anemia, dangerous drug–drug interactions and renal function decline. By leveraging artificial intelligence, the system can detect subtle patterns in longitudinal clinical data, such as hemoglobin trends or estimated glomerular filtration rate (eGFR) trajectories, that may precede adverse events [16,17]. Through automated alerts generated by predefined thresholds or AI-based algorithms, clinicians can be notified of early signs of deterioration and initiate timely diagnostic or therapeutic interventions. This model shifts the paradigm from patient-initiated concern to system-generated risk recognition, aligning with broader goals of predictive, preventive, and personalized medicine [18].

A secondary objective is to optimize healthcare resource utilization by reducing unnecessary in-person visits, duplicative testing, and emergency department admissions. By promoting remote, algorithm-driven decision-making, the model facilitates a more efficient allocation of resources while preserving patient safety and continuity of care. Additionally, the integration of AI-based risk stratification may support regional health authorities in refining therapeutic plan renewals and directing specialist evaluations to patients with the highest clinical priority [19].

The proposed platform envisions a digital interface accessible to general practitioners, specialists, pharmacists, and patients. It would centralize relevant clinical and laboratory information and incorporate intelligent features such as trend recognition, interaction checkers, and clinical event triaging. Unlike current systems that depend heavily on user input or manual review, this model emphasizes automated alerts and minimal interaction thresholds to enhance clinical oversight in a scalable and sustainable way [20].

Ultimately, by coupling AI with existing regional platforms and real-world prescribing practices, the system aims to improve safety, personalization, and efficiency in DOAC therapy management, particularly for elderly, fragile, or clinically complex patients who are most vulnerable to complications.

An AI-driven remote monitoring system that integrates longitudinal laboratory and clinical data with automated alerts can improve the safety of chronic DOAC therapy by enabling earlier detection of occult bleeding, renal function decline, and high-risk drug interactions

### 1.3. Current Challenges in DOAC Management

The widespread adoption of direct oral anticoagulants (DOACs) has led to a common misconception that they require little or no monitoring. While DOACs do not necessitate regular INR testing like vitamin K antagonists, they are not risk-free, especially in elderly patients, those with impaired renal function, or individuals with multiple comorbidities and polypharmacy regimens [21,22].

In clinical practice, laboratory testing such as anti-Xa levels, thrombin time, or direct DOAC plasma levels is generally reserved for acute situations, such as life-threatening bleeding or emergency surgery. These tools are seldom used for routine outpatient monitoring due to limited availability, high costs, and lack of integration with prescribing systems.

At the same time, emerging real-world evidence highlights a substantial number of patients on long-term DOAC therapy who develop clinically significant anemia or progressive renal impairment without overt symptoms. These complications often remain undetected until they lead to serious clinical consequences, such as hospitalization or transfusion [23,24].

According to the International Society on Thrombosis and Haemostasis (ISTH), a hemoglobin drop of ≥2 g/dL or the need for two or more units of blood qualifies as a major bleeding event, even in the absence of visible blood loss [8,9]. A significant proportion of such events are occult in nature and require complex diagnostic workups, including abdominal ultrasound, endoscopy, and CT angiography. However, these services are not always accessible through primary care or outpatient channels, leading patients to seek urgent evaluation in emergency departments [25,26].

Furthermore, the lack of intelligent digital infrastructure connecting laboratory data, patient-reported outcomes, and prescribing platforms impairs physicians’ ability to make fully informed decisions. Prescriptions are frequently renewed without access to real-time clinical data or risk reassessment, particularly when physicians operate outside hospital networks or rely on fragmented platforms. These limitations not only jeopardize patient safety but also increase the risk of inappropriate continuation of therapy in the setting of deteriorating renal function or rising bleeding risk [13].

In addition, current digital systems do not typically include built-in tools for evaluating drug interactions, cumulative comorbidity burden, or dynamic changes in bleeding or thrombotic risk. Nor do they provide automated alerts or predictive analytics that could assist physicians in identifying emerging clinical risks before they manifest as acute events [27].

Taken together, these limitations create a critical need for a smarter, more integrated model of care, one that moves beyond basic data entry or episodic specialist oversight and instead embraces artificial intelligence to continuously analyze patient data, generate risk alerts, and support early clinical decision-making.

## 2. Methods

### 2.1. Proposal for a Remote Monitoring System

At the core of our proposal is the development of a remote monitoring system for patients on chronic DOAC therapy, centered on artificial intelligence and automatic alert functionalities. Unlike traditional telemedicine tools, which often rely on passive data collection and user-initiated interactions, this model leverages predictive algorithms to detect risk patterns, streamline communication among providers, and trigger early clinical interventions.

The platform would operate as a web-based and mobile-accessible interface, integrated with electronic health records (EHRs) and regional prescribing systems such as SANIARP and SINFONIA. Its primary aim is to continuously evaluate key clinical data and identify patients at increased risk of bleeding, renal dysfunction, or drug interactions, based not only on threshold values but also on longitudinal trends and composite risk indicators.

In the proposed model, predefined cut-offs are established for key laboratory and clinical parameters to trigger automated alerts. For example, a haemoglobin drop greater than 2 g/dL, a progressive decline in estimated glomerular filtration rate (eGFR) below 45 mL/min, or a downward platelet trend generate early-warning notifications for clinicians. Conversely, absolute-risk alerts are activated when values reach critical thresholds, such as pre-dialytic renal function (eGFR < 30 mL/min), severe anaemia or progressive reduction in hemoglobin levels to consecutive follow up, or confirmed thrombocytopenia, prompting immediate physician review and clinical action. These predefined limits ensure that the alert system maintains both sensitivity for early detection and specificity for urgent conditions, while the final clinical assessment remains under full human control.

**Figure 1:**
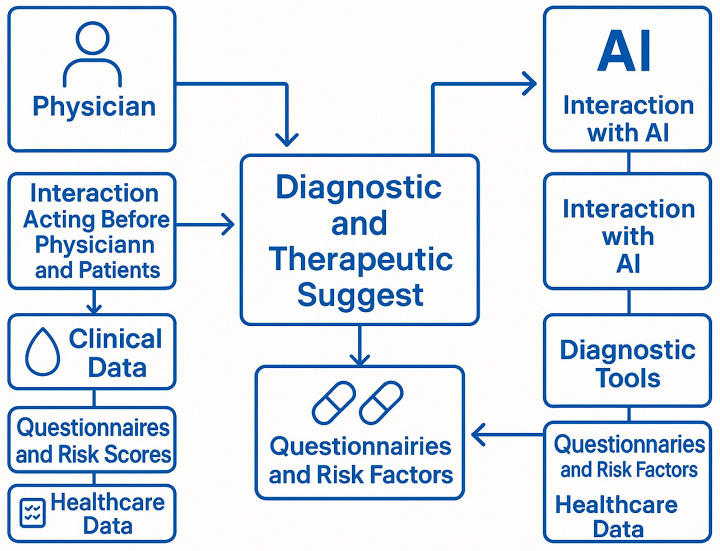
Integrated process through which both physicians and artificial intelligence (AI) contribute to diagnostic and therapeutic suggestions for patients undergoing chronic treatment. Legend: Physician: The human healthcare provider responsible for evaluating patient conditions and making clinical decisions based on experience, data interpretation, and patient interaction; Interaction Acting Before Physician and Patients: Refers to the physician-patient relationship, where clinical judgment and empathy guide decisions prior to algorithmic input; Clinical Data: Includes laboratory values (e.g., hemoglobin, creatinine), vital signs, and other measurable indicators collected from the patient; Questionnaires and Risk Scores: Patient-reported outcomes, standardized bleeding/thrombotic risk scores (e.g., HAS-BLED, CHA_2_DS_2_-VASc), and symptom assessments; Healthcare Data: Administrative, diagnostic, and therapeutic data derived from electronic health records (EHRs) and health system databases; AI: Artificial intelligence platform capable of processing large volumes of structured and unstructured health data to assist in predictive analytics and decision support; Interaction with AI: Refers to the computational processes and machine learning algorithms used to analyse trends, detect risks, and generate clinical suggestions; Diagnostic Tools: Includes algorithms, models, and software modules used by AI to interpret clinical data and generate preliminary assessments; Questionnaires and Risk Factors (AI side): Digitally captured patient inputs and structured risk indicators that feed into AI models for predictive analysis; Diagnostic and Therapeutic Suggest: The central outcome of the integrated process, representing a synthesis of human clinical judgment and AI-generated recommendations. Arrows indicate the bidirectional flow of data and influence, showing that both physician and AI contribute independently and interactively to the generation of therapeutic strategies.

The proposed system should include the following core functionalities (Table 1):

- Scheduled Laboratory Monitoring: Automatic prompts for routine testing of hemoglobin, creatinine, ferritin, and urinalysis every 3–4 months. The platform should enable users to upload original laboratory reports to preserve contextual information. The overall workflow of this integrated model is illustrated in Figure 1, which summarizes how clinical data, patient-reported outcomes, and AI-generated alerts interact to support timely clinical decisions.
- Trend Visualization and AI-Based Alerts: Dynamic visualization of clinical trends (e.g., hemoglobin and eGFR), supported by AI-driven algorithms that classify values into stable, warning, or critical zones using color-coded graphics. The system should generate *automated alerts* when predefined thresholds are crossed or when downward trends suggest clinical deterioration. However, predefined relevant items to review the ongoing treatment are summarized in Table 2.
- Clinical Event Detection: Periodic questionnaires or symptom tracking modules for patients to report fatigue, dizziness, hematuria, melena, or other subtle symptoms that may indicate occult bleeding or renal decline. Alerts are generated automatically based on cumulative risk scoring
- Drug Interaction Monitoring: A built-in interaction checker that scans newly prescribed medications for potential adverse interactions with DOACs, including NSAIDs, antiplatelet agents, and P-gp/CYP3A4 modulators. AI tools can prioritize alerts based on interaction severity and patient comorbidities
- Shared Access for Healthcare Providers: A unified dashboard accessible to general practitioners, internists, cardiologists, hematologists, and pharmacists, enabling coordinated follow-up and therapeutic plan updates. Clinicians can receive real-time notifications of patient alerts and access longitudinal data for clinical decision-making [20].
- Event-Triggered Prioritization: The system can flag patients with recent clinical events, such as emergency department visits, significant lab value changes, or new diagnoses, as requiring urgent review or reassessment. This feature enhances triage and reduces unnecessary delays in care.

Unlike models that rely solely on patient engagement, the proposed system automates much of the risk stratification process through machine learning algorithms. These tools learn from aggregate patient data to refine thresholds, predict adverse events, and guide personalized treatment pathways. By embedding intelligence directly into routine monitoring, the system creates a proactive safety net that complements clinician expertise and supports timely, data-informed care. The AI framework would incorporate supervised learning algorithms such as logistic regression, decision trees, or random forest models to stratify risk based on longitudinal laboratory data (e.g., haemoglobin, creatinine/eGFR), drug prescriptions, and patient-reported outcomes. In addition, unsupervised clustering methods may identify latent risk trajectories across populations. Algorithmic performance would be refined through real-world dataset training and validation.

### 2.2. Data Collection, Processing, and AI Framework

Data collection: integration of structured laboratory data (haemoglobin, creatinine/eGFR, ferritin, urinalysis), prescription records, and patient-reported outcomes via digital questionnaires.

Preprocessing: automated validation of data completeness, outlier detection, and normalization of laboratory values across different laboratories.

Aggregation: consolidation of longitudinal data from heterogeneous sources (regional prescribing platforms, EHRs, laboratory information systems) into a unified patient timeline.

Visualization: dynamic dashboards with colour-coded trajectories (stable, warning, critical) for haemoglobin and renal function, trend graphs, and risk stratification panels.

AI/ML methods: candidate approaches include supervised learning (logistic regression, decision trees, random forest, gradient boosting) for prediction of adverse events, and unsupervised learning (k-means, hierarchical clustering) for identifying latent risk groups. Continuous retraining with real-world datasets ensures adaptability.

Advantages over existing systems: unlike current digital platforms that are mainly administrative, our framework enables predictive, automated, and real-time risk detection, with multi-parameter aggregation and clinician-facing dashboards.

The AI framework employs both supervised and unsupervised learning approaches. Supervised algorithms (logistic regression, random forest, gradient boosting) will be trained to predict adverse events such as occult bleeding or renal decline, while unsupervised clustering methods (k-means, hierarchical clustering) will identify latent patient risk profiles. Data sources include structured laboratory datasets, prescription records, and patient-reported outcomes integrated from regional digital platforms and hospital EHRs. Model validation will rely on real-world datasets with cross-validation, retraining cycles, and accuracy benchmarking to ensure transparency and reproducibility.

### 2.3. Perspective to Have Personalized Adapted Prescription Models

With these predefined alerts, physicians may be prompt to early identification of patients more at risk to develop acute adverse events and by this way a right clinical decision-making joins periodic administrative reassessment for prescription renewals improving long term surveillance of this clinical setting.

## 3. Results

### 3.1. Expected Clinical and Organizational Benefits

From a clinical perspective, the integration of artificial intelligence into DOAC monitoring offers significant potential to improve patient outcomes. By leveraging automated alerts and trend-based analytics, the system can identify early signs of occult bleeding or progressive renal dysfunction, such as subtle declines in hemoglobin or estimated glomerular filtration rate (eGFR), that may otherwise go unnoticed [1,2,7]. Early detection allows for timely interventions including dose adjustment, temporary drug withdrawal, or targeted diagnostic evaluation, thereby reducing the risk of major adverse events and emergency transfusions [8,9,10]. In this way, AI-driven surveillance acts as a safety net, bridging the gap between routine follow-up and the dynamic clinical changes that often precede acute decompensation, including not only overt bleeding events, but also occult haemorrhages, which are frequently under-recognized and may present solely through progressive laboratory changes. AI-driven surveillance acts as a safety net, bridging the gap between routine follow-up and the dynamic clinical changes that often precede acute decompensation, including not only overt bleeding events, but also occult haemorrhages, which are frequently under-recognized and may present solely through progressive laboratory changes.

In elderly patients with chronic conditions who are frequently on long-term anticoagulant therapy, regular monitoring of renal function parameters enables the identification not only of those who fall outside approved therapeutic thresholds, such as having an estimated glomerular filtration rate (eGFR) below 30 mL/min, but also of individuals at risk of evolving toward pre-dialytic or dialytic renal complications within a clinically actionable timeframe.

The AI-driven platform also facilitates earlier recognition of underlying gastrointestinal disorders, such as angiodysplasia, ulcers, or early-stage malignancies, that might present as chronic anemia in anticoagulated patients. By flagging these cases before acute decompensation occurs, the system enables clinicians to arrange elective diagnostic procedures and improve long-term outcomes.

From an organizational standpoint, the proposed model supports a shift toward structured, outpatient-based care. Patients flagged by the system as at-risk can be scheduled for non-urgent diagnostic evaluations such as abdominal ultrasound, endoscopy, or angio-CT, reducing unnecessary emergency department use and optimizing diagnostic workflows [18]. This transformation supports broader healthcare system goals of efficiency, cost containment, and value-based care [19,25].

In terms of resource allocation, fewer emergency visits, transfusions, and hospitalizations translate into direct cost savings and more rational use of healthcare personnel. Nursing and medical staff can be redirected from reactive crisis management to preventive, longitudinal care delivery. Moreover, administrative processes become more efficient as digital platforms streamline documentation, prescription renewals, and inter-provider communication [16,17].

By automating data collection and clinical triage, the system can help reduce care disparities and improve access to timely interventions, particularly for patients in remote or underserved regions. The integration of AI into existing prescribing platforms also strengthens clinical governance, enabling health systems to audit outcomes, monitor adherence, and continually refine care pathways using real-world data.

Ultimately, the combination of automated alerts, trend recognition, and collaborative access fosters a new standard in anticoagulation management, one that is preventive, personalized, and digitally integrated.

Expected measurable outcomes of the proposed framework include a reduction in major and clinically relevant non-major bleeding events, improved patient adherence and follow-up continuity, lower rates of avoidable emergency visits and hospitalizations, and enhanced cost-effectiveness through early detection and preventive management. These parameters will serve as the main performance indicators in future validation studies.

### 3.2. Potential Barriers and Implementation Strategies

Despite its clinical promise, the implementation of remote monitoring of long term treatments with DOACs integrate with an AI-driven supports faces several potential barriers. These include digital literacy gaps among both patients and healthcare providers, variability in regional IT infrastructure, and concerns related to data privacy, especially in systems involving automated decision-making.

A key challenge is ensuring that patients, particularly the elderly and those with limited technological experience, are able to engage with the system in a meaningful way. Although the proposed platform minimizes the need for active user input through automated data analysis and alerts, some degree of patient interaction is still required for symptom reporting, test uploading, or responding to AI-generated questionnaires. To address this, the rollout should include targeted education and training initiatives, simplified user interfaces, and family or caregiver support mechanisms [26,27].

On the provider side, clinicians may initially resist integrating AI-based alerts into their workflow due to concerns about false positives, over-alerting, or the clinical validity of algorithmic recommendations. These concerns can be mitigated through transparent algorithm design, continuous model training with local data, and the inclusion of override or validation functions that maintain physician control over decision-making [13].

Infrastructure and interoperability also present hurdles. For the system to function optimally, it must integrate with regional platforms such as SANIARP and SINFONIA, as well as with electronic health records (EHRs) and laboratory information systems. This requires investment in digital infrastructure, standardized data formats, and collaboration among software developers, health authorities, and providers.

Another critical area is data protection. Since AI models often process sensitive health data, full compliance with GDPR (General Data Protection Regulation) and national privacy regulations is essential. The platform should adopt advanced encryption protocols, role-based access control, and data anonymization for non-clinical uses. Continuous audits and cybersecurity assessments are necessary to ensure the long-term integrity and safety of the system [17,18].

To facilitate adoption, we propose initiating pilot programs in regions with high DOAC usage and existing digital infrastructure. These pilots would test the platform’s real-world performance, gather user feedback, and refine algorithmic thresholds based on local patient populations. Successful pilots could then serve as scalable templates for national deployment.

Policy-level support is also essential. Reimbursement models must evolve to recognize the value of digital and AI-enhanced care models, and national guidelines should incorporate structured remote monitoring as part of DOAC management standards. Furthermore, integrating patient-reported outcome tools, such as the SAMANTA score for menstrual bleeding in younger women, could enhance personalization and promote patient-centered care.

Interoperability represents a key prerequisite for the implementation of the proposed model. Integration with hospital EHRs and national e-prescription platforms would enable seamless data exchange, automatic laboratory data updates, and synchronized therapeutic plan renewals. This requires the adoption of standardized data formats (e.g., HL7 FHIR), secure API connections, and cooperation among regional health authorities, IT providers, and clinical stakeholders.

In summary, while challenges exist, they are surmountable through thoughtful design, stakeholder engagement, and iterative implementation. Artificial intelligence can serve not only as a clinical tool but also as a catalyst for system-wide innovation in anticoagulation management.

## 4. Discussion

Remote monitoring of chronic therapies represents a rapidly evolving frontier in healthcare innovation, particularly in the management of high-risk medications such as direct oral anticoagulants (DOACs). While DOACs offer significant clinical advantages over vitamin K antagonists, their long-term safety depends on regular assessment of bleeding and thrombotic risks, especially in elderly, comorbid, and polymedicated patients [21,23].

In this context, the integration of artificial intelligence into monitoring systems offers a transformative approach. AI can identify patients at elevated risk by analyzing patterns across multiple parameters, such as progressive anemia, renal function decline, and new drug prescriptions, before those risks manifest as acute clinical events. These predictive capabilities enhance the timeliness and precision of clinical decision-making, helping to avoid complications and unnecessary hospitalizations.

Our model is grounded in real-world clinical experience in Southern Italy, where care fragmentation and limited specialist access often result in delayed recognition of DOAC-related complications [15]. By connecting disparate systems, prescription authorization platforms, laboratory databases, and symptom tracking tools, our proposed AI-powered platform addresses a long-standing gap in continuity of care for anticoagulated patients.

The ability to generate automated alerts in response to early clinical signals adds significant value. Rather than waiting for patients to report symptoms or for providers to manually review laboratory results, the system proactively notifies clinicians when intervention may be needed. This supports earlier diagnostic evaluations, treatment modifications, or referrals, and reduces the reliance on emergency services for issues that could be managed electively.

Moreover, the system promotes care standardization and equitable access. For example, patients living in remote or underserved regions, who might otherwise lack timely specialist follow-up, can benefit from the same level of surveillance as those in tertiary centers. By embedding AI into routine care pathways, healthcare systems can achieve both scalability and personalization.

From a public health standpoint, this digital transformation aligns with broader goals of sustainable, value-based care. As healthcare systems face increasing pressure to contain costs while improving outcomes, tools that support early intervention, reduce preventable events, and optimize resource use are indispensable [2,7,15]. AI-driven monitoring offers an efficient solution that leverages technology not just to document care, but to improve it.

Unlike conventional telemedicine models that rely primarily on patient engagement or administrative checks, our hypothesis emphasises predictive analytics and automated alert generation. This proactive approach enables earlier detection of clinically relevant patterns, such as declining haemoglobin or renal function, before symptomatic deterioration occurs, representing a substantial advancement over existing frameworks.

Accordingly, the proposed AI framework operates as a hybrid rule-based decision-support system rather than a fully autonomous predictive tool. The algorithm identifies deviations beyond pre-established cut-offs—such as marked haemoglobin reduction, renal function deterioration, or platelet decline—and issues corresponding alerts. However, clinical validation, diagnostic interpretation, and therapeutic decisions remain the exclusive responsibility of physicians, ensuring human oversight and ethical compliance in all steps of patient management.

Similar successful implementations of artificial intelligence have already been reported in cardiology, where predictive and imaging-based models have improved diagnosis, monitoring, and individualized care. For instance, a recent study demonstrated the effectiveness of AI algorithms in cardiac imaging and arrhythmia prediction, enhancing clinical decision-making and patient [28]. These experiences confirm that AI integration can strengthen both diagnostic precision and proactive patient management, supporting the applicability of our proposed DOAC monitoring model within a broader cardiovascular and digital health framework.

Legal and ethical considerations must also be addressed. The deployment of AI in clinical practice requires transparency, validation, and oversight. Algorithms must be continually updated and trained on diverse populations to avoid biases and maintain accuracy. Nonetheless, when responsibly implemented, AI offers an opportunity to augment clinical practice and improve safety in ways that traditional systems cannot achieve alone.

From an ethical and technical perspective, the proposed AI-supported framework adheres to key principles of explainability, transparency, and bias mitigation. The system is designed to be interpretable by clinicians, allowing the logic behind each alert to be traced and validated (“explainable AI”). Regular auditing and model retraining are foreseen to minimize algorithmic drift and ensure that predictions remain accurate and equitable across diverse patient populations. Furthermore, the human-in-the-loop structure guarantees that all automated outputs are reviewed by physicians before any clinical action is taken. This approach not only preserves clinical responsibility but also promotes fairness, trustworthiness, and compliance with emerging European standards on AI governance and ethical use in medicine.

In accordance with the European Union’s emerging regulatory framework, the proposed model adheres to the principles outlined in the European AI Act and in the EMA guidance on artificial intelligence in medicinal products and medical devices. These documents highlight the need for transparency, risk management, and human oversight throughout the AI lifecycle. The model therefore ensures compliance with the General Data Protection Regulation (GDPR) through secure data anonymisation, encryption, and restricted access protocols. Moreover, the system maintains a human-in-the-loop structure whereby physicians validate all AI-generated alerts before any clinical action is undertaken. This governance framework ensures accountability, protects patient privacy, and aligns the proposed platform with European standards of ethical and responsible AI use in healthcare.

In conclusion, remote monitoring systems that incorporate artificial intelligence and automated alerts represent a logical and necessary evolution in the chronic management of patients on DOACs. This model can improve safety, streamline care delivery, and serve as a blueprint for broader digital health innovations across chronic disease domains.

This manuscript presents a conceptual framework derived from real-world clinical workflows and existing regional digital infrastructures, rather than validated experimental data. Pilot testing and retrospective validation are planned as subsequent phases to assess the system’s feasibility, predictive accuracy, and clinical effectiveness under real-world conditions.

## 5. Conclusions

Chronic treatment with DOACs requires more than periodic prescription renewals; it demands continuous and proactive clinical oversight. In the absence of structured follow-up, even the most advanced anticoagulants can lead to avoidable complications such as occult bleeding, renal deterioration, or inappropriate treatment continuation.

The integration of artificial intelligence into DOAC management represents a strategic advancement in patient safety and healthcare efficiency. By leveraging automated alerts, AI-driven risk prediction, and real-time trend analysis, the proposed remote monitoring model enables timely clinical interventions and supports personalized therapeutic adjustments. This system not only enhances individual patient outcomes but also alleviates pressure on emergency departments and hospital resources by reducing unplanned admissions and unnecessary diagnostics.

Implementing such a model will require coordinated efforts among clinicians, digital health developers, administrators, and policymakers. However, the potential to transform anticoagulation care, making it more responsive, intelligent, and sustainable—justifies the investment.

Ultimately, the future of chronic anticoagulant therapy lies not solely in pharmacological innovation, but in how effectively we can harness digital tools and artificial intelligence to deliver safer, smarter, and more equitable care. This model of integration between artificial intelligence and pre-programmed clinical alerts represents a meaningful step toward tailored medicine, enabling more precise and proactive management strategies that are responsive to each patient’s evolving clinical profile.

**Figure 2:**
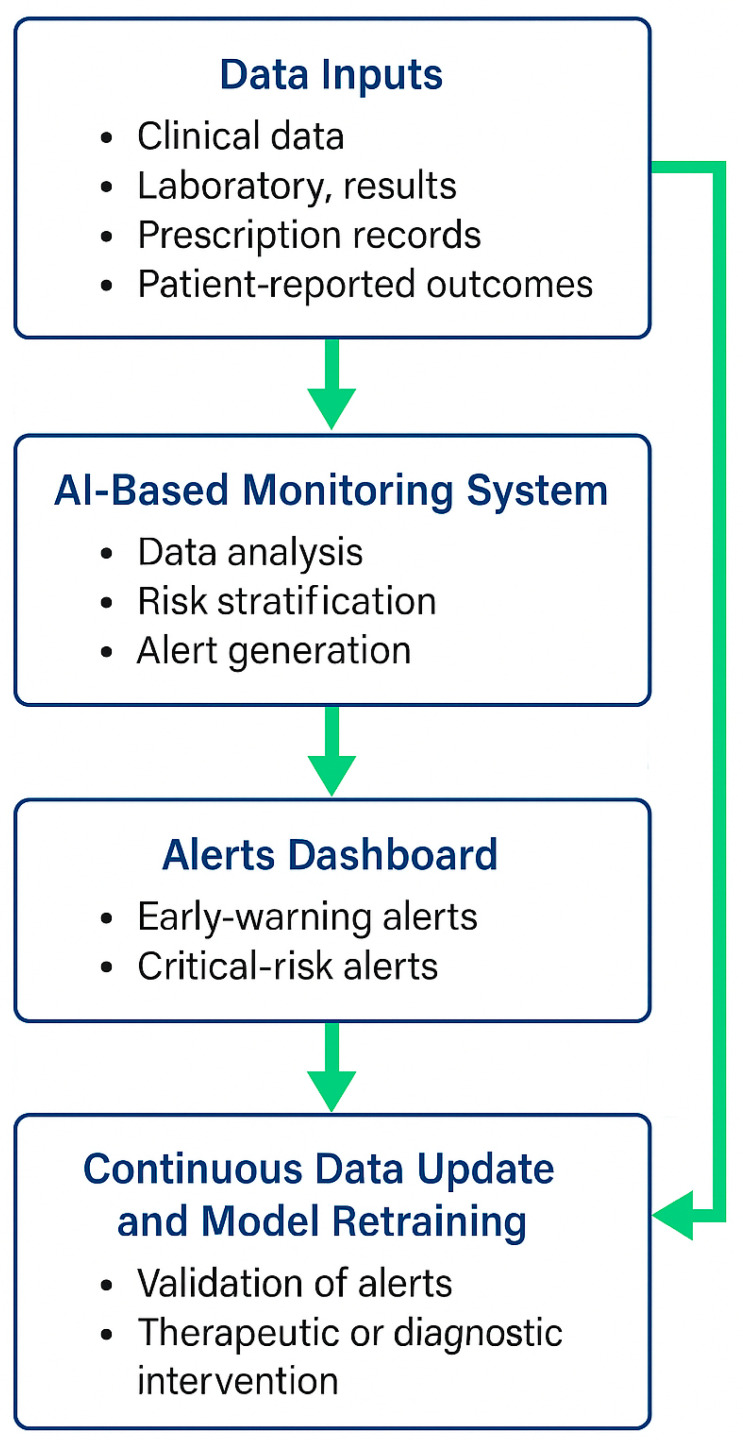
Workflow of the AI-Based DOAC Monitoring System. Legend: The diagram illustrates the proposed workflow of an artificial intelligence (AI)-supported system designed for the continuous monitoring of patients receiving direct oral anticoagulants (DOACs). Clinical, laboratory, prescription, and patient-reported data are collected and processed within the AI-based monitoring platform, which performs data analysis, risk stratification, and alert generation. Alerts are categorised as early-warning or critical-risk and displayed in the clinician dashboard for review. Physicians validate the alerts, undertake therapeutic or diagnostic interventions when necessary, and feed updated patient data back into the system. This closed-loop process enables continuous data refinement and model retraining, ensuring that the algorithm remains accurate, transparent, and clinically reliable.

**Figure 3:**
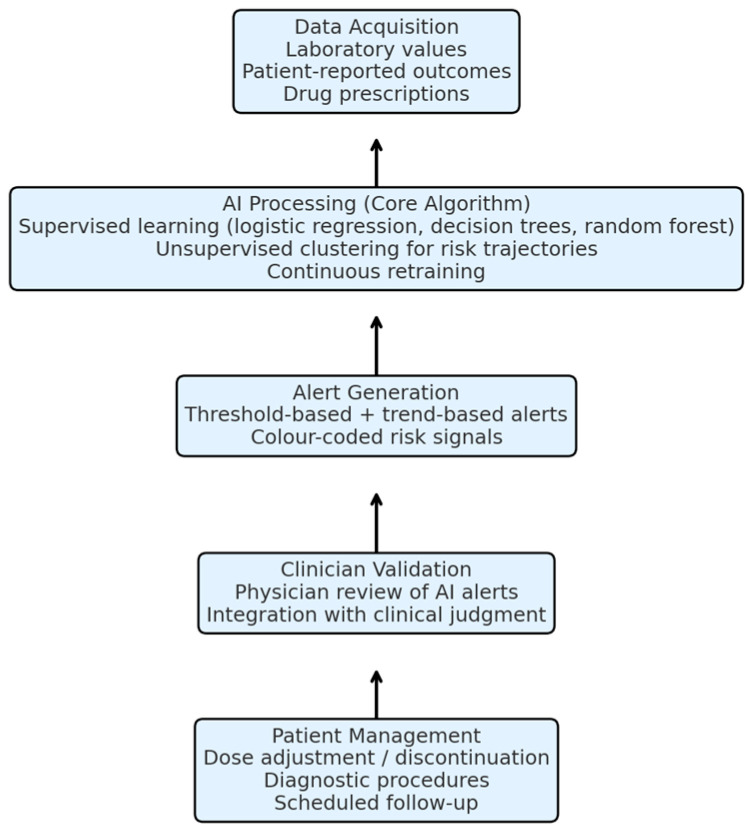
Core algorithm and working schema of the proposed AI-based monitoring model. The system integrates laboratory values, patient-reported outcomes, and prescription data. AI algorithms analyse these parameters, generate predictive alerts, and provide clinicians with actionable insights, who then validate and apply them to guide patient management.

This hypothesis posits that by integrating structured laboratory and clinical data into an AI-driven monitoring system, predictive alerts can be generated to support timely clinician intervention, thereby reducing preventable complications in patients treated with DOACs. This working hypothesis is summarised in Figure 2 and Figure 3, which illustrates the core algorithm and operational workflow of the proposed AI-based model.

## Figures and Tables

**Table 1:** Key Components of the Proposed Remote Monitoring System for DOAC Therapy.

Component	Function
Scheduled Laboratory Monitoring	Automated reminders and data entry for periodic tests (e.g., hemoglobin, creatinine, ferritin, urinalysis)
Trend Analysis and Automated Alerts	Visualization of lab value trends with AI-driven alerts based on color-coded risk thresholds
Symptom Surveillance	Periodic patient-reported symptom tracking (e.g., fatigue, bleeding signs, renal-related symptoms) via digital questionnaires
Drug Interaction Monitoring	Real-time alerts for newly prescribed drugs that may interact with DOACs (e.g., NSAIDs, antiplatelets)
Shared Provider Access	Unified dashboard accessible to general practitioners and specialists for coordinated care
Digital Integration with Prescribing Systems	Seamless linkage with platforms like SANIARP and SINFONIA for treatment plan validation and updates

**Table 2:** Predefined Items for AI-Supported Remote Monitoring of Chronic DOAC Therapy.

Parameter	Threshold/Trigger	Suggested Action	Rationale/Notes
Hemoglobin decline	≥1 g/dL in 3 months; ≥2 g/dL; iron deficiency; Ferritin < 30 ng/mL	Review DOAC dose; consider holding DOAC or search for other sources	Detect and prevent occult gastrointestinal blood loss
eGFR decline	≥10 mL/min/1.73 m^2^ or ≥20% drop; eGFR ≤ 45 mL/min/1.73 m^2^; <30 mL/min/1.73 m^2^	Nephrology assessment	Monitor for progressive renal impairment
Platelet count	Drop by ≥25% or <100,000/μL	Review dose adjustments according to drug labels	Avoid excessive DOAC exposure
Creatinine increase	Positive urinalysis or rising creatinine trend	Further renal evaluation	Detect possible renal dysfunction
Drug interactions	Azole antifungals (ketoconazole, itraconazole, posaconazole, voriconazole); antivirals (ritonavir, cobicistat, etc.); antiarrhythmics (amiodarone, dronedarone); macrolides	Manage potential drug–drug interactions	Prevent changes in DOAC activity and bleeding risk

## Data Availability

Authors declare that no new data were created for the present article.

## References

[1] Kaplon S.C., Aqtash S., Gilbride D., Chan C., Farjo R. (2024). Implementation of a Telemedicine Direct Oral Anticoagulant Monitoring Program at a Safety-Net Hospital. Hosp. Pharm..

[2] Ferreira L.B., de Almeida R.L., Arantes A., Abdulazeem H., Weerasekara I., Ferreira L.S.D.N., Messias L.F.d.A., Couto L.S.F., Martins M.A.P., Antunes N.S. (2023). Telemedicine-Based Management of Oral Anticoagulation Therapy: Systematic Review and Meta-analysis. J. Med. Internet Res..

[3] Gardiner C., Williams K., Mackie I.J., Machin S.J., Cohen H. (2006). Can oral anticoagulation be managed using telemedicine and patient self-testing? A pilot study. Clin. Lab. Haematol..

[4] Connolly S.J., Ezekowitz M.D., Yusuf S., Eikelboom J., Oldgren J., Parekh A., Pogue J., Reilly P.A., Themeles E., Varrone J. (2009). Dabigatran versus warfarin in patients with atrial fibrillation. N. Engl. J. Med..

[5] Patel M.R., Mahaffey K.W., Garg J., Pan G., Singer D.E., Hacke W., Breithardt G., Halperin J.L., Hankey G.J., Piccini J.P. (2011). Rivaroxaban versus warfarin in nonvalvular atrial fibrillation. N. Engl. J. Med..

[6] Granger C.B., Alexander J.H., McMurray J.J., Lopes R.D., Hylek E.M., Hanna M., Al-Khalidi H.R., Ansell J., Atar D., Avezum A. (2011). Apixaban versus warfarin in patients with atrial fibrillation. N. Engl. J. Med..

[7] Guede-Fernández F., Silva Pinto T., Semedo H., Vital C., Coelho P., Oliosi M.E., Azevedo S., Dias P., Londral A. (2024). Enhancing postoperative anticoagulation therapy with remote patient monitoring: A pilot crossover trial study to evaluate portable coagulometers and chatbots in cardiac surgery follow-up. Digit. Health.

[8] Schulman S., Angerås U., Bergqvist D., Eriksson B., Lassen M.R., Fisher W., on behalf of Subcommittee on Control of Anticoagulation of the Scientific and Standardization Committee of the International Society on Thrombosis and Haemostasis (2010). Definition of major bleeding in clinical investigations of antihemostatic medicinal products in surgical patients. J. Thromb. Haemost..

[9] Khorsand N., Majeed A., Sarode R., Beyer-Westendorf J., Schulman S., Meijer K., Subcommittee on Control of Anticoagulation (2016). Assessment of effectiveness of major bleeding management: Proposed definitions for effective hemostasis: Communication from the SSC of the ISTH. J. Thromb. Haemost..

[10] Abdoellakhan R.A., Beyer-Westendorf J., Schulman S., Sarode R., Meijer K., Khorsand N. (2019). Method agreement analysis and interobserver reliability of the ISTH proposed definitions for effective hemostasis in management of major bleeding. J. Thromb. Haemost..

[11] D’Errico M.M., Piscitelli P., Mirijello A., Santoliquido M., Salvatori M., Vigna C., Vendemiale G., Lamacchia O., Fontana A., Copetti M. (2022). CHA2DS2-VASc and R2CHA2DS2-VASc scores predict mortality in high cardiovascular risk population. Eur. J. Clin. Investig..

[12] Pisters R., Lane D.A., Nieuwlaat R., de Vos C.B., Crijns H.J., Lip G.Y.H. (2010). A novel user-friendly score (HAS-BLED) to assess 1-year risk of major bleeding in patients with atrial fibrillation: The Euro Heart Survey. Chest.

[13] Vudathaneni V.K.P., Lanke R.B., Mudaliyar M.C., Movva K.V., Kalluri L.M., Boyapati R., Mounika K.L. (2024). The Impact of Telemedicine and Remote Patient Monitoring on Healthcare Delivery: A Comprehensive Evaluation. Cureus.

[14] Treadwell J.R., Jepson C., Ivlev I., Reston J.T. (2023). Reducing Adverse Drug Events Related to Anticoagulant Use in Adults: Rapid Response. Making Healthcare Safer IV: A Continuous Updating of Patient Safety Harms and Practices [Internet].

[15] Shambu S.K., B S.P.S., Gona O.J., Desai N., B M., Madhan R., V R. (2021). Implementation and Evaluation of Virtual Anticoagulation Clinic Care to Provide Incessant Care During COVID-19 Times in an Indian Tertiary Care Teaching Hospital. Front. Cardiovasc. Med..

[16] Ballestri S., Romagnoli E., Arioli D., Coluccio V., Marrazzo A., Athanasiou A., Di Girolamo M., Cappi C., Marietta M., Capitelli M. (2023). Risk and Management of Bleeding Complications with Direct Oral Anticoagulants in Patients with Atrial Fibrillation and Venous Thromboembolism: A Narrative Review. Adv. Ther..

[17] Fujisaki T., Sueta D., Yamamoto E., Buckley C., Sacchi de Camargo Correia G., Aronson J., Tallón de Lara P., Fujisue K., Usuku H., Matsushita K. (2024). Comparing Anticoagulation Strategies for Venous Thromboembolism Associated With Active Cancer: A Systematic Review and Meta-Analysis. JACC CardioOncol..

[18] Barrios V., Cinza-Sanjurjo S., García-Alegría J., Freixa-Pamias R., Llordachs-Marques F., Molina C.A., Santamaría A., Vivas D., Suárez Fernandez C. (2022). Role of telemedicine in the management of oral anticoagulation in atrial fibrillation: A practical clinical approach. Future Cardiol..

[19] Rashedi S., Keykhaei M., Sato A., Steg P.G., Piazza G., Eikelboom J.W., Lopes R.D., Bonaca M.P., Yasuda S., Ogawa H. (2025). Anticoagulation and Antiplatelet Therapy for Atrial Fibrillation and Stable Coronary Disease: Meta-Analysis of Randomized Trials. J. Am. Coll. Cardiol..

[20] Al Ammari M., AlThiab K., AlJohani M., Sultana K., Maklhafi N., AlOnazi H., Maringa A. (2021). Tele-pharmacy Anticoagulation Clinic During COVID-19 Pandemic: Patient Outcomes. Front. Pharmacol..

[21] Pozzi M., Mitchell J., Henaine A.M., Hanna N., Safi O., Henaine R. (2016). International normalized ratio self-testing and self-management: Improving patient outcomes. Vasc. Health Risk Manag..

[22] Heneghan C.J., Garcia-Alamino J.M., Spencer E.A., Ward A.M., Perera R., Bankhead C., Alonso-Coello P., Fitzmaurice D., Mahtani K.R., Onakpoya I.J. (2016). Self-monitoring and self-management of oral anticoagulation. Cochrane Database Syst. Rev..

[23] Walkden J.A., McCullagh P.J., Kernohan W.G. (2019). Patient and carer survey of remote vital sign telemonitoring for self-management of long-term conditions. BMJ Health Care Inform..

[24] Jang I. (2021). A Systematic Review on Mobile Health Applications’ Education Program for Patients Taking Oral Anticoagulants. Int. J. Environ. Res. Public Health.

[25] Barcellona D., Fenu L., Marongiu F. (2017). Point-of-care testing INR: An overview. Clin. Chem. Lab. Med..

[26] Crowther M., Hirsh J. (1997). Low-molecular-weight heparin for the out-of-hospital treatment of venous thrombosis: Rationale and clinical results. Semin. Thromb. Hemost..

[27] Verhaert D.V.M., Betz K., Gawałko M., Hermans A.N.L., Pluymaekers N.A.H.A., van der Velden R.M.J., Philippens S., Vorstermans B., Simons S.O., den Uijl D.W. (2022). A VIRTUAL Sleep Apnoea management pathway For the work-up of Atrial fibrillation patients in a digital Remote Infrastructure: VIRTUAL-SAFARI. Europace.

[28] Cersosimo A., Zito E., Pierucci N., Matteucci A., La Fazia V.M. (2025). A Talk with ChatGPT: The Role of Artificial Intelligence in Shaping the Future of Cardiology and Electrophysiology. J. Pers. Med..

